# Enhancing Functional Rehabilitation Through Orthotic Interventions for Foot and Ankle Conditions: A Narrative Review

**DOI:** 10.7759/cureus.49103

**Published:** 2023-11-20

**Authors:** Momin Mohaddis, Saad A Maqsood, Emmanuel Ago, Sushmit Singh, Zahra Naim, Seema Prasad

**Affiliations:** 1 Trauma and Orthopaedics, Warrington and Halton Hospitals NHS Trust, Liverpool, GBR; 2 General Practice, Shadan Institute of Medical Sciences, Hyderabad, IND

**Keywords:** ankle-foot orthosis, ankle and foot, ankle brace, foot and ankle surgeon, ortho surgery, orthoses, foot & ankle surgery, ankle foot orthoses, lower-limb orthoses, foot orthoses

## Abstract

Non-surgical, conservative approaches to foot and ankle conditions are of important consideration. Orthotics play a significant role in treating these conditions, preventing progression, and alleviating pressure on affected areas, thereby promoting normal gait. This article aims to assess the utility and effectiveness of various orthotic treatments in different clinical scenarios. We reviewed 27 peer-reviewed articles using electronic databases, employing keywords such as "orthoses," "orthotic treatment," "arthritis," "neuropathy," and "foot and ankle trauma." Studies conducted in recent decades have explored the effectiveness of orthoses in various conditions, including connective tissue disorders, tendon and ligament injuries, foot arthritis, neuropathic and inflammatory wounds, and sports-related recurrent injuries. Orthotic management has proven effective across diverse foot and ankle conditions. Integrating orthotic treatment with systemic approaches benefits patients with foot and ankle disorders. We believe this review can be utilised by clinicians in the management of foot and ankle disorders.

## Introduction and background

Foot orthoses have been utilised by physicians for over 150 years in the treatment of musculoskeletal conditions, as well as the prevention of injury and optimisation of lower limb biomechanics [1]. Orthoses are defined as medical devices that support, align, correct deformity, and improve or prevent further deformity [2]. In other words, orthotic devices are prescribed to support the musculoskeletal system by modifying structural or functional ailments associated with certain disease processes [3]. In 2016, the World Health Organisation (WHO) issued a compendium comprising 50 assistive products, encompassing orthoses, orthotic therapeutic footwear, and lower limb prosthetic devices. These were introduced to enhance accessibility to such products [4].

**Figure 1:**
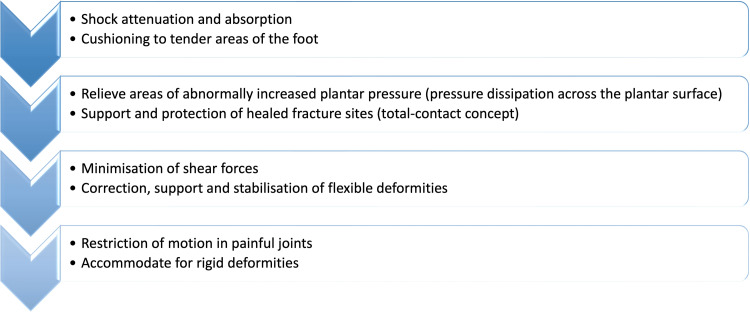
Potential objectives related to the utilisation of foot orthosis according to Janisse DJ Adapted from Janisse DJ; A scientific approach to insole design for the diabetic foot [5]; Image created by the authors.

The overarching objective behind employing foot orthoses lies in the mitigation of foot discomfort while concurrently enhancing mobility and overall quality of life. Janisse DJ (1993) identified eight potential objectives, outlined in Figure 1, related to the utilisation of foot orthosis, as elucidated in the medical literature [5,6].

**Figure 2:**
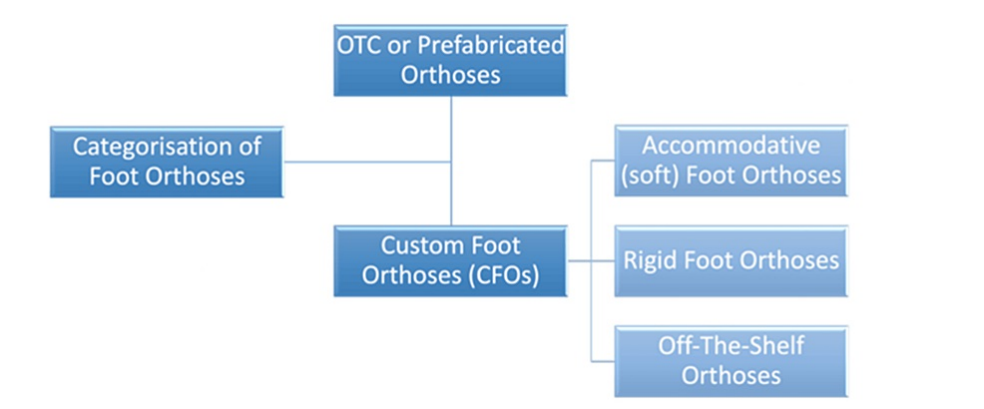
Broad categorisation of foot orthoses Over-the-counter (OTC) or prefabricated orthoses: prescribed by clinicians for multiple ailments, they are standardised, readily available, and relatively cheap; however, they can be less durable. Custom foot orthoses (CFOs): custom-made and individualised, they are more durable but can be expensive [2]. CFOs can be further subdivided into: a. Accommodative (soft) foot orthoses: provide a cushioning effect and act as shock absorbers; they are prescribed for conditions resulting in fixed deformities and insensate feet. b. Rigid foot orthoses: used to control/decrease motion in conditions resulting in flexible deformities. c. Off-the-shelf orthoses: prescribed in conditions resulting in pain and discomfort, without ulcers or neuropathy, to provide cushioning and shock attenuation. Image created by the authors.

Generally, foot orthoses can be divided into two broad categories, as outlined in Figure 2.

Through the modulation of the intensity, positioning, and timing of reaction forces exerted by the lower limbs, foot orthoses facilitate the restoration of natural functionality. The primary locus of supportive action exhibited by orthotic devices is the medial longitudinal arch, through which they mitigate abnormal loading on the foot and lower extremities during weight-bearing activities. This phenomenon is extensively documented in the realm of medical literature [1,7].

In general, pain serves as a commonly encountered symptom in the context of foot and ankle disorders. The presence of pain during periods of rest typically indicates an underlying inflammatory or neurological condition. Conversely, when pain is experienced during weight-bearing activities, it often points to a mechanical disorder [8]. Numerous medical conditions, such as various plantar foot types, excessive knee flexion leading to gait abnormalities in individuals with spastic cerebral palsy, Charcot-Marie-Tooth disease, and rheumatoid arthritis, have garnered attention in multiple studies for showcasing the beneficial effects of orthotic interventions. Furthermore, research has demonstrated the utility of foot orthoses in preserving balance and preventing falls among elderly patients. Consequently, the primary objective of this review article is to enhance comprehension of the mechanisms and efficacy of orthotic tools in diverse clinical scenarios while offering clinicians a multidisciplinary perspective on disease management. This comprehensive approach aims to inspire researchers to embark on more extensive, innovative, and forward-looking investigations in this field.

## Review

Methodology

The results of this review are based on a comprehensive analysis of a total of 27 articles. The selected studies focus primarily on investigating biomechanical modifications associated with various clinical conditions and have undergone rigorous peer review prior to publication. Non-peer-reviewed articles, conference abstracts, commentaries and editorials were excluded. The articles were sourced from electronic databases, including PubMed, MEDLINE, and Google Scholar, where a systematic search was conducted to identify relevant literature.

The keywords "orthoses", "orthotics", "orthotic treatment," "foot and ankle disease", "foot and ankle trauma", "neuropathy", "neuropathic foot", and "arthritis" were employed during the search process using Boolean operators ("AND" and "OR"). We utilised the snowball search method, which entails reviewing references within foundational sources to reveal further pertinent materials. Findings are organised and presented in a structured manner, categorised under distinct subheadings corresponding to various clinical conditions. These subheadings encompass topics such as neuropathic foot, stress fractures, flat foot, and others, providing a comprehensive overview of the insights derived from the published literature.

Results

In accordance with diverse biomechanical requirements, a variety of foot and ankle orthotic designs have been developed to address specific needs. One notable example is the University of California Biomechanics Laboratory (UCBL) orthosis, which is engineered to manage postural flexible deformities by positioning the hind limb in a neutral orientation. Additionally, there are moulded ankle foot orthosis (MAFO) devices intended to restrict ankle range of motion, dynamic AFOs designed to offer proprioceptive feedback, hinged AFOs that deliver dorsiflexion assistance for patients experiencing foot drop, and Arizona-based AFOs tailored for conservative management of tendon dysfunction [6]. Throughout this discussion, we delve into the practical applications of orthotic devices, elucidating their roles in diverse clinical contexts based on a review of the pertinent literature.

Plantar Fasciitis

The plantar fascia is a resilient connective tissue band that provides crucial support to the arch of the foot. Plantar fasciitis, a condition stemming from degenerative changes in the plantar fascia due to overuse stress, is characterised by a classic symptom of sharp heel pain. This ailment is commonly observed in individuals engaged in activities such as running, older adults and those with professions that entail prolonged periods of standing and continuous weight-bearing. Additionally, obesity is a recognised risk factor for the development of plantar fasciitis.

In the initial stages of treatment, conservative and symptomatic approaches are typically employed to alleviate pain. These measures include periodic rest, the application of ice, deep friction massages, and the use of oral and topical nonsteroidal anti-inflammatory drugs (NSAIDs). Orthotic devices and night splints are often prescribed as complementary therapies. In more advanced cases, invasive interventions such as cortisone injections, Botox injections, platelet-rich plasma therapy, and extracorporeal shock wave therapy have shown efficacy. Surgical intervention is considered a last resort for chronic, longstanding cases.

Orthotic utilisation plays a crucial role in the comprehensive treatment plan for plantar fasciitis. Studies indicate that orthotics are highly effective, with an 89% success rate in reducing pain associated with plantar fascia degeneration [9]. The primary objectives of orthotic use are to augment the midfoot contact area and provide medial arch support. These adjustments result in the redistribution of force, alleviating pressure on the heels and thereby reducing strain on the plantar fascia during weight-bearing activities. Two commonly employed types of orthotics are prefabricated viscoelastic heel pads and multi-layered insoles, which have demonstrated effectiveness comparable to that of custom-made orthotics in most cases. However, patients with concurrent plantar fasciitis and structural misalignments such as pes planovalgus or pes cavus may necessitate custom-moulded orthoses for optimal outcomes [10,11].

Tendon Disorders

Orthotic intervention has proven efficacious in the management of several tendon disorders. Below, we outline a selection of these disorders:

Posterior tibial tendon dysfunction (PTTD): The tibialis posterior tendon plays a pivotal role as the primary stabiliser of the medial longitudinal arch and a mid-foot inverter. Throughout the gait cycle, it functions to elevate the arch, creating a rigid structure in the mid-foot and hind foot. When this tendon loses its functionality, often due to traumatic rupture, it can lead to a condition known as adult-acquired flat foot. This condition is often accompanied by deformities, including internal rotation of the tibia, heel valgus alignment, and in advanced cases, lateral shifting of the distal fibula, which results in lateral hindfoot pain [12].

Consequently, the principal objective of orthotic treatment in cases of posterior tibial tendon dysfunction is to alleviate strain on the impaired tendon by restoring the medial arch and mitigating pronation. In the early stages of posterior tibial tendon dysfunction (PTTD), when deformities are still flexible, semirigid orthotic devices are commonly employed. These devices maintain neutral hindfoot alignment and restrict forefoot abduction. Custom articulated ankle-foot orthoses have demonstrated a 77% success rate in conservatively managing the initial two stages of PTTD. However, in later stages, when deformities become more rigid, the approach shifts toward accommodation rather than correction. The overarching goals at this stage are to provide support for the flat foot, alleviate pain, and enhance functional status [13,14].

Achilles tendinopathy: This is a degenerative condition affecting the Achilles tendon, characterised by a multifactorial aetiology. Contributing factors include both extrinsic elements such as acute trauma and intrinsic factors such as vascularity, dysfunction of the gastrocnemius-soleus muscles, age, sex, body weight, and lateral ankle instability. Chronic damage to the Achilles tendon can occur due to repetitive hindfoot motion in the frontal plane and lateral heel striking during pronation [15].

The predominant symptom of Achilles tendinopathy is pain in the tendon during weight-bearing activities. The initial management of this condition typically involves conservative approaches, including rest, medication (either steroidal or nonsteroidal), stretching, strength training, and orthotic adjustments. The primary aim of orthotic treatment is to alleviate strain on the damaged tendon by correcting excessive foot pronation and resultant calcaneal eversion. In cases of insertional Achilles tendinopathy, open-back shoes or Achilles sleeves may be recommended. On the other hand, non-insertional Achilles tendinopathy may require a controlled ankle movement boot to limit tendon mobilisation during the acute phase of degeneration. It is worth noting that the effectiveness of orthotic treatment in acute conditions of the disease is not extensively documented in the literature [16,17].

In chronic cases of Achilles tendinopathy, custom-moulded foot orthotic devices have demonstrated efficacy in randomised controlled trials. Additional tools that prove valuable in managing this condition include heel lifts, ankle joint dorsiflexion night splints, and shoe modifications [18,19]. These interventions collectively contribute to the comprehensive management of Achilles tendinopathy.

Arthritis

Arthritis, characterised by degenerative changes within the joints, is often accompanied by pain, joint dysfunction, and consequent disability. The principal objective of employing orthotic interventions in arthritis is twofold: to mitigate pain and enhance functionality. Orthotics can also serve as a means to postpone the necessity for surgical interventions. Notably, distinct orthotic tools are employed for addressing arthritis in the foot and ankle.

Ankle arthritis:The incidence of ankle arthritis is more common among elderly patients, while in young individuals, it often arises due to repetitive injuries. Evidence-based studies suggest that conservative management is a beneficial approach prior to considering invasive methods. This conservative approach encompasses limiting weight-bearing exercises and transitioning to non-weight-bearing activities such as cycling and swimming. It also involves the use of walking aids and orthotic devices [20].

Ankle arthritis commonly involves painful sagittal plane movement at the tibiotalar joint. Therefore, the primary goal of employing orthotic tools is to restrict excessive dorsiflexion and plantar flexion while maintaining proper gait biomechanics [6]. Moulded ankle-foot orthoses (AFOs) and Arizona braces have been effective in reducing loading on the ankle joint. When combined with a rocker-bottom soled shoe, these tools compensate for limited ankle joint movement by facilitating efficient forward propulsion. Additionally, devices such as a solid ankle cushion heel have demonstrated utility in reducing ankle joint movement, contributing to a smoother gait cycle by enabling a seamless transition from heel strike to toe-off [21]. These orthotic interventions play a crucial role in enhancing the quality of life for individuals dealing with ankle arthritis.

Subtalar arthritis: In cases where arthritis affects the subtalar joint, individuals often experience pain during heel strikes and during eversion and inversion movements. When addressing subtalar arthritis, the primary objective of orthotic interventions is to manage motion in the coronal plane and restrict subtalar eversion and inversion. Several orthotic tools have been studied and found to be effective in treating this condition, with a focus on controlling hindfoot stability.

Two orthotic solutions that have demonstrated efficacy are hinged ankle-foot orthosis (AFO) and supramalleolar orthoses (SMO). The SMO, in particular, extends proximally near the malleoli, offering enhanced hindfoot stability. In cases where both ankle and subtalar joints are affected by arthritis, employing fixed ankle-foot orthosis (AFO) with or without supramalleolar orthoses (SMO) is considered an optimal approach for controlling motion and providing the necessary support [22].

Midfoot arthritis: In midfoot arthritis, which typically results in painful transverse tarsal and tarsometatarsal joints, particularly during dorsiflexion of the foot in the gait cycle, orthotic interventions have proven beneficial. These orthotic tools are designed to provide support and alleviate discomfort. One effective orthotic design involves a device that cups the heel on both sides, medially and laterally, while also offering midfoot arch support. These orthoses are observed to stabilise the midtarsal joint, limiting excessive abduction and adduction of the midfoot. This controlled motion aids in reducing pain associated with midfoot arthritis. Additionally, shoe inserts made from carbon graphite material have demonstrated approximately 75% effectiveness in reducing plantar pressure, further alleviating discomfort and promoting better foot function.

Another noteworthy solution is the use of double-rocker bottom shoes, which have been studied and found to be beneficial in cases of midfoot arthritis. These shoes feature a thin, contoured central portion that reduces pressure on the midfoot without increasing pressure on the forefoot and hindfoot. This mechanical design aids in the seamless transition from heel strike to toe-off during the gait cycle while preventing excessive bending of the foot [23].

Forefoot arthritis: In forefoot arthritis, where the metatarsophalangeal joint is most affected, controlling joint motion is essential for managing the condition. In the initial stages of the disease, effective interventions include the use of stiff-soled footwear along with figure-of-eight taping, which can help limit motion and provide relief.

Orthotic devices specifically designed for forefoot arthritis management have proven highly effective, with an observed success rate of approximately 84% for conservative treatment. These orthotic solutions include Morton extension plates, rocker bottom shoes and metatarsal bars [24].

Neuropathic Foot

Neuropathic foot conditions often result in high-pressure points that can lead to ulcers, particularly in patients with compromised peripheral nervous system function. These conditions are frequently associated with various systemic and neurological disorders, such as diabetes, syphilis, HIV, B12 deficiency, postherpetic neuralgia, alcoholism, autoimmune disorders, and Lyme disease, and hereditary conditions such as Charcot-Marie-Tooth disease and demyelinating polyneuropathy. The areas most susceptible to ulcers are the heels and metatarsal heads, which are prone to callus formation due to high friction [25].

Symptoms associated with neuropathic foot conditions range from hyperkeratotic lesions to foot deformities and limitations in range of motion. Given the multifaceted nature of these complications, a comprehensive treatment plan is essential. This approach involves addressing both systemic issues, such as diabetes management, by physicians and localised treatment by wound care practitioners in collaboration with orthotic specialists.

Orthotic interventions play a vital role in the management of neuropathic foot conditions. The primary goal of orthoses is to offload sensitive areas with pressure transferred to more tolerant areas. Studies have shown varying effectiveness levels, ranging from 20% to 80% [26]. For instance, patients without ulcerations may benefit from long-term maintenance through depth inlay shoes and multi-density accommodative foot orthoses, measures that help to prevent complications and maintain foot health. Whilst in cases of Charcot joint arthropathy or chronic ulcerations, the objectives shift to joint immobilisation and unweighting to protect the affected areas.

**Figure 3:**
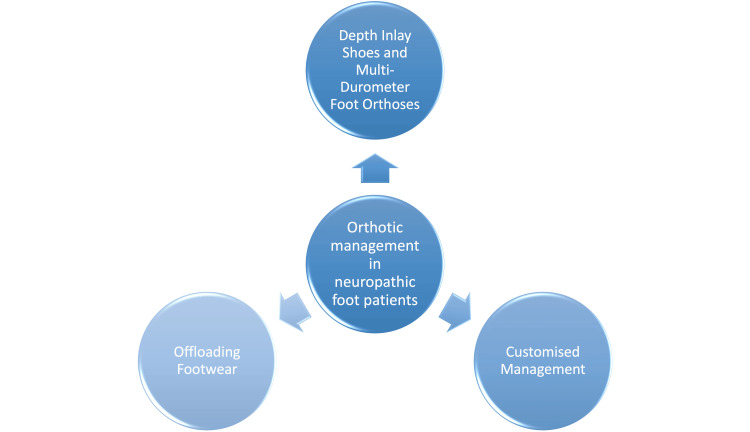
Primary modalities for the orthotic management of neuropathic foot patients 1. Depth Inlay Shoes and Multi-Durometer Foot Orthoses: Depth inlay or diabetic shoes provide protection to the dorsal and plantar aspects of the foot and accommodate multi-durometer foot orthoses. They can be modified with the inclusion of a medial or lateral flare, sole lift, or rocker sole to widen the patient’s base of support, address atypical joint moments, accommodate a leg-length discrepancy, and increase the stance phase timing. These combinations reduce pressure on high-pressure areas and provide better support [27]. 2. Offloading Footwear: Offloading shoes are designed to minimise sheer pressure at wound sites. Forefoot offloading shoes support the metatarsal heads and phalanges, keeping the ankle in a relatively dorsiflexed position. Conversely, hindfoot offloading shoes limit pressure on the calcaneus. Offloading footwear is modifiable with rocker-bottom soles or by removing pre-punched pads [27]. 3. Customised Management: In cases of Charcot's arthropathy, active ulcerations, and severe deformities, custom-made orthotic tools are used. Examples include the Charcot restraint orthotic walker (CROW) orthosis, which provides full support to the plantar aspect of the foot, ankle and calf; the patellar tendon-bearing (PTB) orthosis, applied to the patellar tendon (ligament), popliteal fossa, and medial tibial flare, which provides plantar offloading; and the conventional ankle-foot orthosis (AFO) with moulded calf lacer, which provides unloading of the plantar aspect of the foot by circumferential loading of the calf musculature. These tools provide specific support and offloading tailored to the patient's condition [28]. Image created by the authors.

Three primary modalities, summarised in Figure 3, are used for orthotic management in neuropathic foot patients.

Miscellaneous Disorders

Additionally, orthotic interventions have proven effective in cases of ligament tear injuries, ankle instability, and ankle sprains. Semirigid orthotics or air cast braces are recommended by the American College of Sports Medicine for ankle support, especially for athletes, avid runners, and military personnel, as they can effectively prevent recurrent ankle sprains [29,30].

Planovalgus foot (flat foot): Flat feet can be categorised based on aetiology as either idiopathic, characterised by normal feet with lax joints, or acquired, the more common variant and typically associated with dysfunction of the tibialis posterior tendon [31]. Medial arch support insoles, which redistribute pressure, reduce strain on the tibialis posterior tendon, and improve overall foot mechanics, and shoe modifications, such as extra cushioning and arch support, are used in the conservative management of flat feet. In cases where conservative management fails, surgical decompression of the tendon sheath may be considered a second-line treatment option [32].

Cavorasus foot: Characterised by an elevated medial longitudinal arch and a heel tilted in a varus position, this condition stands in contrast to planovalgus foot disorders. In cases of mild cavus foot, the typical approach involves the utilisation of an insole designed to accommodate the plantarflexed first metatarsal [33]. As a secondary treatment option, surgical interventions may include metatarsal base osteotomy and tendon transfer.

Lesser toe deformities: Often observed in the elderly population, this condition results from a muscular imbalance between intrinsic and extrinsic flexors and extensors. Treatment strategies typically involve a forefoot insole along with footwear featuring deeper toe space with the primary objective being to alleviate pain and minimise interphalangeal joint friction [34].

## Conclusions

Diverse shoe modalities and foot orthotic devices have demonstrated effectiveness in the conservative management of various acute and chronic foot and ankle conditions. These conditions encompass traumatic injuries, congenital abnormalities, arthritis, neuropathy, diabetes, and sports-related injuries. Facilitating interactive sessions between surgeons and orthotic practitioners is imperative to foster collaboration and the exchange of treatment strategies. This collaborative approach ensures that both treatment modalities align to provide optimal patient care. Additionally, patient education plays a pivotal role, as patients need to be informed regarding the advantages and limitations of off-the-shelf orthotic tools and custom-made orthoses. This education empowers patients to make informed decisions about the usage of these tools tailored to their specific clinical needs.
